# Do political relations affect international trade? Evidence from China’s twelve trading partners

**DOI:** 10.1186/s41072-020-00076-w

**Published:** 2020-10-22

**Authors:** Gregory Whitten, Xiaoyi Dai, Simon Fan, Yu Pang

**Affiliations:** 1 Department of Economics, Lingnan University, Hong Kong, China; 2 Corporate Sales Division, China Mobile Hong Kong, Hong Kong, China; 3 School of Business, Macau University of Science and Technology, Macau, China

**Keywords:** International trade, Conflict, Political tensions, China

## Abstract

China’s growing influence on the world has generated profound effects on the political and economic decisions of her partner nations. Recent conflict escalation between China and western countries gives rise to widespread concern over the possibility of delinking China from global trade and supply chain. By drawing on utility theory, we suggest that the political relationship is a key determinant of collective emotions of consumers and trading companies and consequently the interactions between importers and exporters. We hypothesize that warmer relations lead to larger increases (or smaller decreases) in trade while cooler relations have the opposite effect. Based on monthly data of China and her twelve trading partners from 1981 to 2019, our study provides an empirical investigation into the association between political relationship and bilateral trade flows. Our results show that shocks to relations are highly persistent and frequently cause changes in trade. However, relations themselves are little influenced by changes in trade, changes that show little persistence. We also address the US-China trade war and the observation that innovations to China’s exports to the US improve China’s relations with the US while shocks to American exports to China worsen relations from China’s perspective.

## Introduction

Globalization and economic integration is a salient feature of the modern world. In particular, the increasing division of labor and fragmentation of supply chains have led to the globalization of production and shipping of many commodities and services, which has enhanced international economic cooperation. Greater trade flows provide supportive evidence of this cooperation (see Krugman, Obstfeld, and Melitz 2014). Against this trend of globalization, however, international politics is not necessarily becoming more cooperative, an idea that had been promoted in the popular press and academia (Friedman 2000, Mandelbaum 2003, Witt 2019). Political tensions may considerably hinder all aspects of economic integration such as trade, investment, supply chain, and transport logistics.

China presents herself as an interesting case for considering the link between international conflict and international trade. Since 1949, China has experienced relations both excellent and tense with other countries, often switching quickly. While worsening relations have led to severe armed conflicts in certain cases (e.g., the Sino-Indian war of the 1960s, the Sino-Vietnamese war of the 1970s), most cases of tense international relations between China and other countries have not led to explicit military aggression. Over the past four decades, China has substantially increased its exports and imports of manufactures and services and has emerged in the global marketplace with greater economic significance and more prominent political influence (Lukin 2019). Meanwhile, China has often been the target of criticism in some Western media for not strictly following international norms. These criticisms tend to generate a heated reply from the Chinese mainstream media. One may wonder if outright military aggression or a general worsening of diplomatic relations hinders international trade. Investigating the links between China’s political and economic influences emerges as a reasonable line of inquiry.

This paper is motivated by the recent association of falling trade and political conflicts between the US and China. In the context of escalating tensions over aggressive rhetoric concerning the South China Sea and accusations of espionage and intellectual property theft, the Trump administration released the new National Security Strategy in 2017, which described China as a revisionist power aiming to “*shape a world antithetical to (the US) interests and values.*”^1^ Poor diplomatic relations have emerged in tandem with greater calls to limit trade activities (Cheng, Whitten, and Hua 2020). The COVID-19 pandemic outbreak in 2020 seems to have pushed bilateral relations to the breaking point. Many American companies are shifting their supply chains to other Asian countries, and China’s exports to the US are decreasing sharply (Bown 2019). Both the general public and political leaders have expressed their fears of decoupling of Sino-US economic ties given their increasing deterioration of political relations:^2^

*Nowadays, it is common to hear arguments warning of a split of the global economy into mutually exclusive American and Chinese spheres of influence... [I]f indeed we arrive at a fully ‘decoupled world’, it would herald the return of an ‘iron curtain’ between the East and the West, and “the beginning of a new conventional and nuclear arms race with all its attendant strategic instability and risk.”*

Motivated by these anecdotal observations, this paper aims to conduct an empirical study addressing how does China’s political relation with a foreign country affect their bilateral trade.

Although the above discussion emphasizes the causality of politics on trade, recent history shows the potential for causality to go the other way. Trade has historically served as a lighting rod of political disputes (Hiscox 2002). It is expected that closer economic integration helps to reduce international conflicts. For instance, China’s relations with the West soured after the 1989 Tiananmen Square Incident, prompting a fall in international trade and investment (Brantstetter and Lardy 2008). Then-candidate Bill Clinton promised to exclude China from the to-be-formed World Trade Organization (WTO) owing to displeasure with China’s human rights record. Unexpectedly, President Clinton changed his mind, believing that greater trade could improve Sino-US relations as well as Chinese relations with the world generally (Martin and Roberts 2017).

This paper adds to empirical studies on the interactions between international political relations and trade. Due to scarcity of data on political relations, the existing empirical research remains scanty, with most qualitative analyses being in the literature of political science (e.g., Pollins 1989, Schneider, Barbieri, and Gleditsch 2003). This literature essentially investigates how institutions affect trade. In the economics literature, Moenius and Berkowitz (2011) present a more general analysis about how institutions influence international trade. Glick and Taylor (2002), Che, Du, Lu, and Tao (2015), and He, Nielsson, and Wang (2017) are three empirical studies that are particularly related to this paper. Based on the gravity model of trade, Glick and Taylor (2002) demonstrate that over the period 1870-1997, the wars between two countries have substantial and persistent impacts on their trade. Che, Du, Lu, and Tao (2015) show that in China, regions that suffered more civilian casualties during the Japanese invasion from 1937 to 1945 tend to trade less with Japan in recent decades, *ceteris paribus*. He, Nielsson, and Wang (2017) find that non-violent events harming the Cross-Strait relations adversely affect stock prices of pro-independence Taiwanese firms that are economically exposed to mainland China via either investments or exports.

In this paper, we focus on international conflicts generally, which are mainly emotional in nature and do not involve the potential destruction of factors of production, rather than wars exclusively. We examine the impacts of the change of political relations between China and twelve trading partners, namely, Australia, Germany, France, India, Indonesia, Japan, Korea (South), Pakistan, Russia, the United Kingdom, the United States, and Vietnam. Our results from pair-by-pair analyses (e.g., China and one of the preceding countries) show that trade, political relations, and gross domestic product (GDP) all exist in a long-run stable relationship as evidence by the finding of at least one cointegrating vector for the aforementioned variables.

We present impulse response functions, showing that the largest effect of a positive to shock to each our three variables of interest (political relations, China’s exports to a partner, a partner’s exports to China) is nearly always on the corresponding variable. Yet the persistence of these effects differs. Shocks to political relations are notably persistent while shocks to exports (be it from China or from a partner) dissipate often within a year. A positive shock to relations usually leads to greater trade flows between China and the economies (especially for Australia, France, Germany, Indonesia, Pakistan, the UK) though trade declines in a few cases (India and Vietnam). A positive shock to partner exports to China results in better relations for six countries (Australia, France, Germany, India, Japan, Vietnam); interestingly, the same shock leads to slightly or significantly worse relations (from China’s perspective) for the remaining 6 country pairs (Australia, Indonesia, Korea, Russia, the UK, the US). A positive shock to China’s exports to a partner boosts China’s relations with that partner for a few countries (Indonesia, Korea, the UK, the US) and lowers relations for a few others (India, Japan, Vietnam).

Moreover, our Granger causality tests show that relations frequently Granger-cause trade in both directions while trade Granger-causes less frequently. Our analysis sheds some light on the ongoing shifts in trade flows and global sourcing in response to the changing political and diplomatic climate. A VAR model that incorporates 11 of the 12 country pairs shows that China’s trade with any country rarely depends on China’s political relations with a third country.

The structure of this paper is as follows. “Theoretical background” section provides the theoretical underpinnings to our conjecture. “Methodology” section describes our statistical methodology. “Data” section discusses the data used. “Results” section presents estimation result. “Conclusion and further discussion” section concludes.

## Theoretical background

The potential effect of political conflicts on international trade can be rationalized by the utility theory, which is developed to study multiple dimensions that determine people’s decision-making. In economics, the concept of utility can be interpreted as an index of happiness and satisfaction. Conventional wisdom holds that an individual can obtain utility from several sources such as product quantity, diversity, brand, and perceived quality based on their preferences. Elster (1998) and Glaeser (2005) provide surveys on the literature that incorporates emotions in economic theories, and explores the impacts of emotional factors on social and business behaviors. They postulate that an individual’s utility sources from not only material consumption but also emotions.

In psychology literature, collective emotions are defined as emotions that are shared by a large fraction of individuals in a certain society, are evident in social gatherings, and are an important feature of nationalism (e.g., Bar-Tal, Halperin, and De Rivera 2007, Von Scheve and Ismer 2007). Collective emotions are usually contagious in the sense that one’s emotions and behaviors directly cause similar emotions and behaviors in other people. Glaeser (2005) develops a model of rational public choice, in which hatred is fostered by interactions between some politicians’ supply of hate-creating stories and voters’ willingness to listen. By this logic, a change in home country’s political relation with a foreign country can be a significant trigger of domestic citizens’ collective emotions toward from that country, and consequently, emotions enter into these utility-maximizing decisions of stakeholders at every level, including consumers, exporters, and importers (Pollins 1989).

In the absence of international conflicts, nationalist emotions are at a low ebb. Consequently, consumers tend to focus only on conventional dimensions such as quantity and quality. However, in the presence of an exogenous international conflict, nationalism may suddenly flare up from sensational media coverage. Many people may refuse to purchase commodities and services produced by the “enemy” country. For instance, the 2012 conflict between China and Japan over a group of islands known in Chinese as Diaoyu and in Japanese as Senkaku led to boycotts by each nation of the other nation’s products^3^. In contrast, a more harmonious international relationship will foster globalization and incur fewer boycotts.

Finally, trade barriers, both tariff-based and non-tariff-based, are detrimental to economic efficiency. For example, the literature of supply chain management addresses the capacity and efficiency of transport logistics in promoting foreign trade (Hausman 2004, Lai, Ngai, and Cheng 2004, Lai and Cheng 2009, Munim and Schramm 2018). Poor port infrastructure and backward shipping and communication technologies constitute a non-tariff barrier for many firms in the evolution of international trade (Nordås, Pinali, and Grosso 2006, Lai, Pang, Wong, Lun, and Ng 2012). Similarly, poor political relationship can be regarded as an effective non-tariff trade barrier. Racial hatred, mutual distrust, unresolved historical tensions, and longstanding political disagreement drive two countries apart, thereby presenting a hidden transaction cost or even an insurmountable obstacle for them to build economic interconnections. In contrast, warm relationship or similar ideology helps to enhance friendly feelings between two peoples, which in turn facilitates the development of closer business ties, as manifested in their increasing trade, shipping, and economic cooperation.

## Methodology

### Model specification

A model such as a gravity equation is used traditionally to explain bilateral trade for all pairs of countries in a given set of countries where trade between countries *i* and *j* depends on *i*-specific variables, *j*-specific variables, and (*i*,*j*)-specific variables (Head and Mayer 2014). The question of interest for this paper (the importance of political relationships in explaining Chinese trade) makes a gravity equation unfeasible owing to the lack of data quantifying political relationships between non-Chinese countries. For example, a gravity model of bilateral trade for all pairs of a set of countries with a political relations variable as a control would require observations on the political relations between any and all pairs, (*i*,*j*), of countries (e.g., the US and Japan). As we have measure of bilateral political relations only for pairs of countries where China is one of the members (i.e., no data on changes in political relations between the US and Japan), the gravity framework is not directly applicable. The lack of political relations data for country pairs not involving China also limits our ability to control for 3 ^*rd*^-country effects on China?s political relations and trade. One salient example is that former US Senator Sam Nunn claimed that stability on the Korean peninsula was an important motivation for accepting China into the WTO, which leads to greater US-Chinese trade (Martin and Roberts 2017).

In the absence of a particular functional form to follow for our estimating equation, we use a general functional form informed by gravity (as it includes GDP as a key determinant for trade). Head and Mayer (2014) detail a long literature of gravity models that explain bilateral trade as depending contemporaneously on the GDP of the exporting and importing economies. The literature study bilateral trade over time with repeated cross-sections of the simple gravity model (Glick and Rose 2002, 2016) or dynamically with lagged levels of GDP (Olivero and Yotov 2012).

The gravity literature provides strong justification to model trade, GDP, and political relations with dynamic models, namely Vector Autoregression (VAR) and Vector Error Correction Models (VECM). VAR modeling captures the linear interdependencies among multiple time series, allowing for more than one evolving variable. VECM modeling allows for the possibility that the variables in question exist in a long-run equilibrium where exogenous deviations in any one variable lead to the restoration of the long-run equilibrium via changes in the other variables; the magnitude of the deviation determines the magnitude of the adjustment speed in pursuit of restoring the long-run relationship.

We first adopt a simple five-equation model to study each of the country pairs (China, *i*, and a corresponding nation, *j*). Let *X*_*ijt*_ denote the real value of aggregate exports from *i* to *j* at time *t* and let Political_*ijt*_ denote the political relation score between *i* and *j* at time *t*. Our model to study an individual country pair is of the following form:

$$\begin{array}{@{}rcl@{}} X_{ijt} &=& \sum_{l=1}^{\Lambda} \alpha_{1ijl}L^{l}.X_{ijt} + \sum_{l=1}^{\Lambda} \beta_{1ijl}L^{l}.X_{jit} + \sum_{l=1}^{\Lambda} \gamma_{1ijl}L^{l}.{GDP}_{it} + \sum_{l=1}^{\Lambda} \delta_{1ijl}L^{l}.{GDP}_{jt} \\ && + \sum_{l=1}^{\Lambda} \xi_{1ijl}\text{Political}_{ijt} + \epsilon_{1t}\\ X_{jit} &=& \sum_{l=1}^{\Lambda} \alpha_{2ijl}L^{l}.X_{ijt} + \sum_{l=1}^{\Lambda} \beta_{2ijl}L^{l}.X_{jit} + \sum_{l=1}^{\Lambda} \gamma_{2ijl}L^{l}.{GDP}_{it} + \sum_{l=1}^{\Lambda} \delta_{2ijl}L^{l}.{GDP}_{jt} \\ && + \sum_{l=1}^{\Lambda} \xi_{2ijl}\text{Political}_{ijt} + \epsilon_{2t}\\ {GDP}_{it} &=& \sum_{l=1}^{\Lambda} \alpha_{3ijl}L^{l}.X_{ijt} + \sum_{l=1}^{\Lambda} \beta_{3ijl}L^{l}.X_{jit} + \sum_{l=1}^{\Lambda} \gamma_{3ijl}L^{l}.{GDP}_{it} + \sum_{l=1}^{\Lambda} \delta_{3ijl}L^{l}.{GDP}_{jt} \\ && + \sum_{l=1}^{\Lambda} \xi_{3ijl}\text{Political}_{ijt} + \epsilon_{3t}\\ {GDP}_{jt} &=& \sum_{l=1}^{\Lambda} \alpha_{4ijl}L^{l}.X_{ijt} + \sum_{l=1}^{\Lambda} \beta_{4ijl}L^{l}.X_{jit} + \sum_{l=1}^{\Lambda} \gamma_{4ijl}L^{l}.{GDP}_{it} + \sum_{l=1}^{\Lambda} \delta_{4ijl}L^{l}.{GDP}_{jt} \\ && + \sum_{l=1}^{\Lambda} \xi_{4ijl}\text{Political}_{ijt} + \epsilon_{4t}\\ \text{Political}_{ijt} &=& \sum_{l=1}^{\Lambda} \alpha_{5ijl}L^{l}.X_{ijt} + \sum_{l=1}^{\Lambda} \beta_{5ijl}L^{l}.X_{jit} + \sum_{l=1}^{\Lambda} \gamma_{5ijl}L^{l}.{GDP}_{it} + \sum_{l=1}^{\Lambda} \delta_{5ijl}L^{l}.{GDP}_{jt} \\ &&+ \sum_{l=1}^{\Lambda} \xi_{5ijl}\text{Political}_{ijt} + \epsilon_{5t} \end{array} $$

where *Λ* is the lag length of the system, all *α*,*β*,*γ*,*δ*, and *ξ* are coefficients to be estimated, and *ε* is the error term. Note that this specification does not address the possibility for trade or political relations between China and country *j* to affect trade or relations between China and country *k* for *j*≠*k*. To take into account the 3rd-country effect, we later adopt a richer model where we try to include all country pairs in our VAR model.

### Estimation technique

Brantstetter and Lardy (2008) document, among others, the tremendous growth of China’s international trade, particularly after the ascendance of Deng Xiaoping in the late 1970s and China’s adherence to the WTO in 2001, and the importance of this growth for China’s economic output. The near-unceasing growth in China’s trade (especially exports but also imports) all but guarantees that recorded values of trade and GDP lack mean-reverting behavior. China’s relations with other countries (e.g., Australia, Germany, Pakistan, South Korea) also demonstrate strong, upward-growing behavior without any mean reversion.

To avoid estimates potentially biased by dynamic behavior in the data, we adopt the following approach based on Berkowitz, DeJong, and Husted (1998) and Whitten (2018) in order to discover a meaningful relationship between international political relations and trade. First, we use Stata’s varsoc command in order to find an appropriate number of lags (as determined by the number of lags that minimizes the Schwarz Bayes Information Criterion or SBIC) for a VAR model in question. Second, we use the vecrank command to apply Johansen’s test for the presence of cointegrating vectors based on a VAR with the number of lags identified in the first step.

In the event that we find at least one cointegrating vector, the third step is to estimate a vector error correction model (VECM) in Stata using the lag length identified by varsoc and the number of cointegrating vectors identified by vecrank and to estimate Impulse Response Functions (IRFs). Since we do not have an a priori model to justify a particular Cholefsky decomposition of the variance-covariance matrix (i.e., imposing restrictions on certain error terms), we estimate ordinary IRFs, not orthogonalized IRFs. The presence of a least one cointegrating vector from the third step demonstrates that a long-run, stable relationship exists among the five variables. Such a long-run relationship implies that we can conduct Granger causality tests on the levels of the variables, which is our final step. If the second step yields no cointegrating vectors, then we estimate a VAR on the variables in differences and conduct Granger causality tests based on those results.

## Data

To perform the regression analysis, we use monthly data on trade, political relations, GDP, and price indices. We create real trade data denominated in US dollars by deflating nominal values of export in domestic currency (converted with exchange rate data) by a price index, where export data are from the IMF’s Direction of Trade Statistics and data on price index and exchange rate are from the IMF’s International Financial Statistics. There are four available price indices, namely Consumer Price Index (CPI) with all items, harmonized CPI, CPI of retail prices, and Producer Price Index (PPI). Not all countries have all price indices available for on a monthly basis, but all price indices are highly correlated. We therefore average the available values across all indices to impute an inflation measure for a particular country in a particular month. GDP data are taken from the IMF’s International Financial Statistics. Note that some data such as price indices and GDP require interpolation from quarterly data (or in the case of Pakistan and Vietnam, from yearly data) to create monthly series. The observations cover the period 1981-2019, with different trading partners varying in timespan due to data availability.

Political relations data come from the *Relationship between China and Major Nations Database* compiled by the Institute of International Relations of Tsinghua University^4^. Let us briefly summarize the construction and interpretation of this dataset as follows. On a monthly basis, Tsinghua researchers evaluate each influential event concerning China’s foreign relations reported by *People’s Daily* and China’s Ministry of Foreign Affairs and then calculate a score for each relationship. In other words, the relations are all evaluated from China’s perspective. The scores range from −9 to 9, with 0.1 being the smallest increment. A higher score means a better bilateral relationship.

As the numbers reported in the political relations data come without specific units of measurement, the question of interpreting these numbers as cardinal as opposed arises. The technical documentation provided by Tsinghua demonstrates that their methodology for constructing the index yields a cardinal measure of relations and that all measures can be comparable across country pairs and over time. Consider the following three examples:^5^

**Example 1:** Given that the Sino-US relation score in March 2004 is 1.2, the Sino-US relation score in April 2004 can be calculated in the following five steps.

1. Collect information on the relevant news occurring between April 1st to 30th.
2. The positive news in this month, which, according to Tsinghua’s evaluation, has a impact of 2.6. Meanwhile, negative news generates a impact of −1.9.
3. Apply the formula developed of the research team to compute the positive factor equals (9−1.2)/9×2.6=2.3 and the negative factor equals (9+1.2)/9×(−1.9)=−2.2. Therefore, the net value is 2.3+(−2.2)=0.1.
4. All events occurring in April improve the relation by 0.1/5=0.02, where 5 is a constant according to the formula. Since 0.02 is smaller than 0.1 (the smallest increment), the score is rounded down.
5. Calculate the score for April 2004 as 1.2+0=1.2,

**Example 2**: From May to June 1989, the Sino-US relation score falls from 2.6 to 0.4, decreasing by 2.2. From April to May 1999, the Sino-US relation score falls from 1.9 to −0.3, decreasing by 2.2. It can be inferred that the change rate in May-June 1989 is the same as that in April-May 1999.

**Example 3**: If the China-Russia relation score and the China-Vietnam relation score are both 3 in any given month, then China’s relation with Russia is equally good as her relation with Vietnam. In May 1989, the Sino-US relation is rated as 2.6, while the Sino-Japan relation is rated as 4.3. The difference is 1.7.

Another concern might arise as the possibility of endogeneity in the event that the news cited in the above excerpts includes announcements about international trade negotiations. We argue based on the evidence provided in the document that endogeneity is not a concern owing to the focus in the construction of the index on issues that are almost exclusively political or military in nature. The technical document does not state explicitly the types of events that it uses but the examples provided to describe its process include war, military conflict, the establishment of diplomatic relations, state visits, multilateral summits among leaders, the Tiananmen Square Incident, conflits over historical issues, maritime rights, United Nations (UN) reforms, nuclear energy and weapon issues, and human right issues. The document mentions international trade once on its page 7:

Take the Sino-Japanese relation in 2005 as an example. Economic ties between the two countries have continued to develop, and their trade volume has increased by 9.9%; however, they have great controversies on historical issues, maritime rights issues, and UN reforms. In the bilateral relations, security and political relations are high-level politics, while economic and cultural relations are low-level politics. Therefore, the Sino-Japanese relation in 2005 is assessed as being disharmony^6^.

Therefore, we contend that endoegeneity is not a problem when using the political relations data to study China’s international trade or vice versa.

**Fig. 1:**
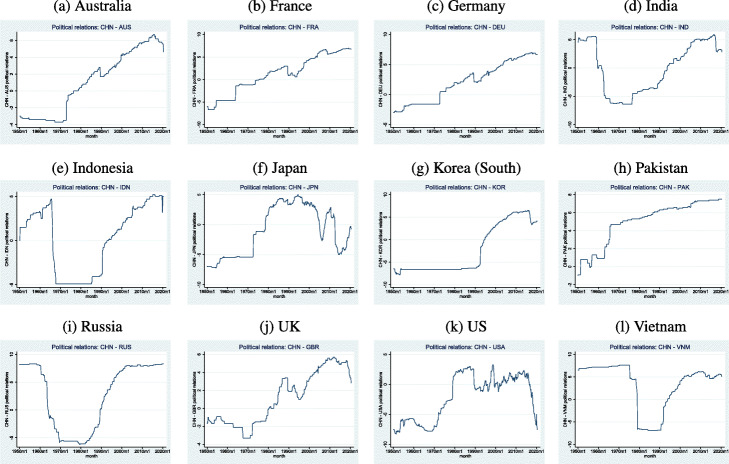
Trends of the Scores of Political Relationship between China and Its Twelve Trading Partners

Figure 1 plots the scores of political relationships between China and her twelve trading partners over time. A key event in China’s foreign relations often corresponds to the movement in the relation. For example, the relation scores for developed countries (e.g., Australia, Germany, France, Japan, the UK, the US) drop in mid-1989 when the Tiananmen Square Incident occurred. Country-specific events manifest themselves as well. As China established formal ties with Australia in 1972, their relation scores increased noticeably. There are sharp falls in the scores of relationship between China and France in the 1990s when French planned to sell weapons to Taiwan and in 2008 when the French president at the time, Nicolas Sarkozy, met the Dalai Lama^7^. A declining value of Sino-British relationships in the late 1960s corresponds to incidents such as riots in Hong Kong and attacks on British and Chinese diplomatic missions, but the score increases substantially after the signing of the Sino-British Joint Declaration regarding Hong Kong^8^. The Sino-Japanese relation improved in the 1970s and early 1980s, coinciding with formal recognition of China by Japan (MOFA 1972). Declines in the relation score take place simultaneously with statements made regarding Taiwan and with conflicts between Japan and China regarding the Diaoyu/Senkaku Islands (MOFA 2005)^9^.

Let us proceed to discuss the scores for developing countries. The relation measure between China and Indonesia mostly increased through the 1950s and early 1960s and fell dramatically, coinciding with a failed Communist coup in Indonesia and the Indonesian government’s decision to sever relations with China. The restoration of these relations in the late 1980s is indicated by a sharp increase in the relation score (Ku 2002). The Sino-Indian war in the 1960s and India’s nuclear tests in the late 1990s correspond to decreasing relation scores (Maxwell 1972)^10^. The Sino-Vietnamese war leads to the slump in the Chinese-Vietnamese relation score, reversed only by the normalization of relations in the early 1990s (Womack 2006).

## Results

### Test for cointegration

The results from the VECM yield at least 1 (and sometimes more than 1) cointegrating vector for all of our country pairs (see appendix 1). These results imply that political relationships, bilateral trade, and the GDP of the partner countries have a long-run, stable relationship. The Johansen method to test for cointegration will impose coefficients of 1 or 0 on certain variables when calculating the cointegrating vector. Consequently, these coefficients have no standard errors. However, the coefficients for remaining variables are generally significant. Exceptions include the cointegrating vectors for Germany and Indonesia. The coefficients for a given cointegrating vector (including the constant term) are a mixture of positive and negative numbers, as is to be expected. Since the trade and GDP variables generally grow over time and the relation variables (with some exceptions) have positive trends, a vector of coefficients whose dot product with the dynamically-increasing variables that yield a constant number over time must contain some negative numbers.

As cointegration exists for all country pairs, we compute IRFs for them based on the VECM results. Since the cointegrating vectors lack standard errors for some variables, we cannot compute confidence intervals for the IRFs. Consequently, our analysis is limited to the point estimate of the IRFs.

### Impulse response functions

#### Shocks to political relations

**Fig. 2:**
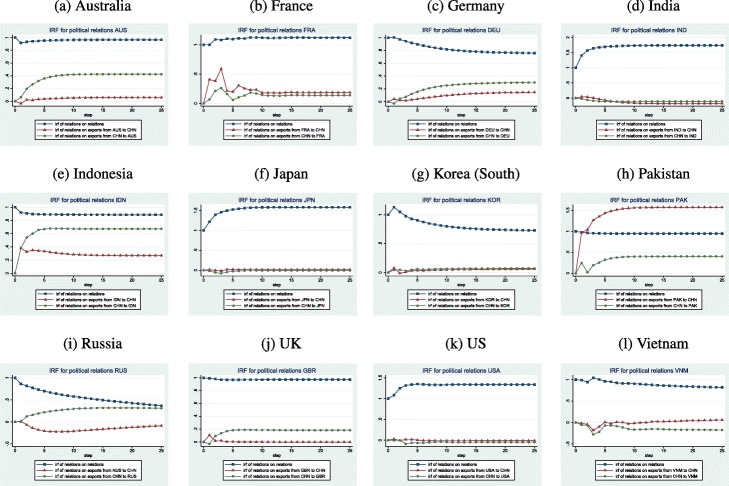
IRFs for Political Relations

Figure 2 provides a visual presentation of IRFs for political relations. A common feature is that the effects are quite persistent and lead to point estimates that are generally persistently higher than are the effects on international trade. A positive shock to relations usually has a larger effect on China’s exports to partner than on the partner’s exports to China (the case for Australia, Germany, Indonesia, Russia, and the UK). A shock to Chinese-Australian relations leads to persistently higher relations and persistently higher trade. Chinese exports to Australia are more responsive than are Australian exports to China. A positive shock to Chinese-German relations leads to a slight improvement after the first month during which Chinese exports to Germany fall slightly but German exports to China rise slightly. Yet, by the next month, all 3 variables change direction: relations fall steadily, Chinese exports to Germany increase steadily, and German exports to China fall before rising again (but not as much as do Chinese exports to Germany). Chinese-Indonesian relations and trade achieve persistently high levels as a result of a positive shock. Chinese exports to Indonesia respond more strongly than do exports from Indonesia to China. A shock to Chinese-Russian relations fades steadily after the first month though relations remain improved even after 2 years. Chinese exports to Russia grow as a result while Russian exports to China fall and recover gradually. The effect of a shock on Chinese-British relations have a similar effect though the growth in British exports to China is short-lived.

In four out of twelve cases, the effect of a positive political shock is almost equally negligible for both types of trade (India, Japan, Korea, the US). A positive shock leads to much better relations between China and India though exports between the economies both shrink over time and remain low for up to 2 years. A similar pattern holds for China and Japan though the decline in trade is less pronounced. An improvement to Chinese-Korean relations falls steadily after 1 month while bilateral trade fluctuates slightly in the first 3 months before achieving a slightly higher level than previously. Improved Chinese-American relations are quite persistent but have little effect on trade. If anything, Chinese exports to the US fall initially and recover only gradually. American exports to China show little response to a shock to relations.

We finally discuss the cases for Vietnam, France, and Pakistan. A positive shock to Chinese-Vietnamese relations oddly causes those relations to fluctuate slightly around the new, higher level, then decline gradually. Contemporaneously, trade between the two economies falls noticeably and then rise. However, while Vietnamese exports to China continue to grow (if slowly), Chinese exports to Vietnam continue to fall (if slowly) in response to the shock to relations. A positive shock to Chinese-French relations leads to persistently higher relations for the next 2 years while trade between the economies also increases. Yet, while the change in political relations is largely monotonic, the trade response to a political shock is more complicated. French exports to China display initially some cyclical behavior (growing, falling, growing) before reaching a steady and higher level about 10 months after the shock to political relations. Chinese exports to France demonstrate similar if more muted behavior. A positive shock to Chinese-Pakistani relations creates a persistent improvement while Pakistani exports to China expand tremendously. Chinese exports to Pakistan also grow (after some initial fluctuations) and remain elevated for the 2 year period.

Overall, the shocks to relations are persistent showing little evidence of fading even after 2 years (Germany, Korea, and Russia are exceptions to this pattern). The response of Chinese exports to the partner country is noticeably larger than is the response of partner exports to China for 5 of the 12 pairs.

#### Shocks to partner exports to China

**Fig. 3:**
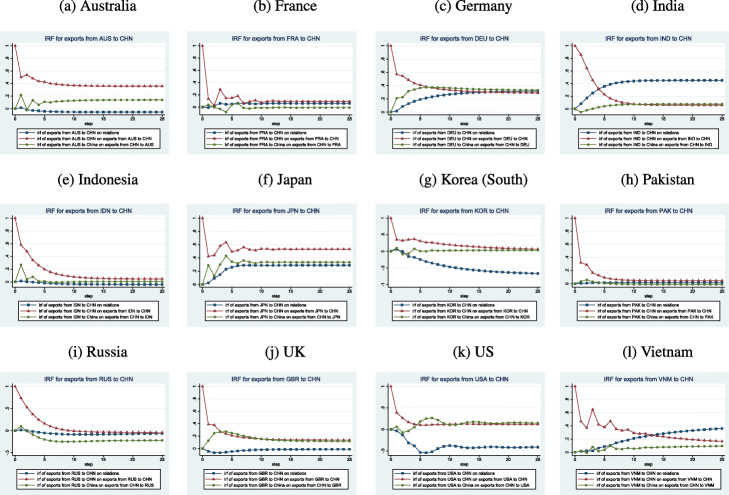
IRFs for Exports from Partners to China

In Fig. 3, we plot the IRFs for a shock on partner exports to China. They generally have the longest-lived effects on partner exports to China, though the magnitude of this effect falls quickly after the innovation. In a slight majority of cases, the next largest effect is on China’s exports to the partner followed by the effect on relations.

This pattern holds for the US, for example, where China’s exports to the US fall briefly following a positive shock to American exports to China before rising. Interestingly, the positive shock to American exports to China leads to a persistent decline in US-China relations. Similar to the US case, a positive shock to Korean exports to China leads to a worsening of Chinese-Korean relations. Chinese exports barely change while Korean exports to China decline after experiencing a shock and return to their original level after 2 years. Russian exports to China increase and then decline steadily after they experience a positive shock. Any positive effect disappears within a year, and exports fall below their previous level. Similarly, relations between the 2 countries worsen and Chinese exports to Russia decline noticeably.

A positive shock to Vietnamese exports to China is generally short-lived and leads to oscillating behavior for the first 7 months before declining steadily over the remainder of 2 years. However, this shock leads to a steady increase in Vietnamese-Chinese relations and a steady if smaller increase in Chinese exports to Vietnam. A positive shock to Japanese exports to China is also transient but converges more quickly to a persistently higher level. Chinese exports to Japan as well as relations improve to a persistently higher level rather quickly (after 6-7 months). The Australian case resembles the Japanese case though relations deteriorates following a positive innovation to Australian exports to China. The innovation to British exports to China is also short-lived though Chinese exports to Britain do rise. Relations worsen slightly before reversing that trend.

Pakistani exports to China decline quite quickly after a positive shock though they converge to a slightly higher level than previously. Relations and Chinese exports to Pakistan change very slightly (the former improve while the latter worsen). A similar pattern happens for Indonesia. The Chinese-Indian relation to a positive shock to Indian exports to China resembles the Chinese Pakistani case. The positive shock falls steadily and converges to a slightly higher level after 9 months. Chinese exports to India remain improved for the duration of 2 years after a slight fall. Relations improve steadily and remain so after around 6-7 months. German exports to China also decline quickly after a positive shock but remain elevated over the 2-year period. Both relations and Chinese exports improve steadily. A positive shock to French exports to China dissipates quickly and somewhat noisily, leading to a slightly higher level after 6-7 months. Relations improve slightly while Chinese exports to France show little movement.

#### Shocks to China’s exports to partners

**Fig. 4:**
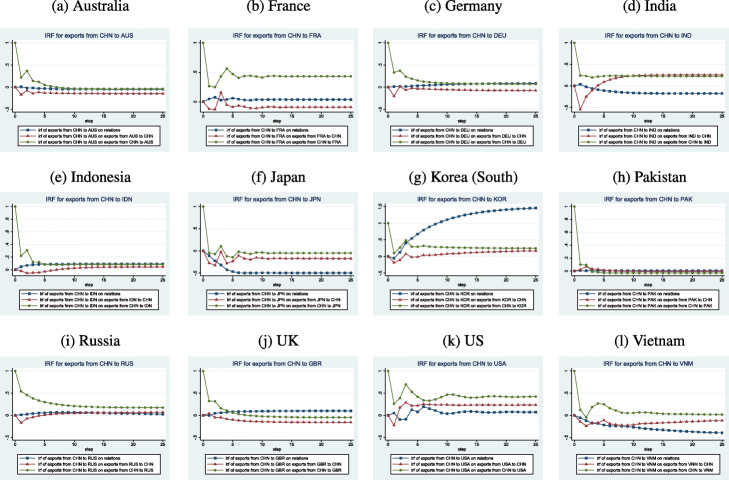
IRFs for Exports from China to Partners

Figure 4 plots the IRFs for exports from China to her trading partners. The strongest response to a positive shock to Chinese exports to a partner economy generally manifests itself on Chinese exports to a partner economy. The next-strongest effect is on relations for half of the pairs and on partner exports to China for the remaining half. As is the case with innovations to partner exports to China, the effect of the innovation on its own series declines quickly before reaching a persistent level that may or may not be the initial level before the shock.

An innovation to Chinese exports to the US oscillates for up to a year before evening out. Relations and American exports to China also oscillate for 5 to 11 months before they all reach higher-than-initial levels, though the effect on relations is modest. Similarly, the positive shock to Chinese exports to Vietnam also decline quickly, rebound, then decline again before dissipating after a year. Both relations and Vietnamese exports to China decline and remain below their initial levels for the 2-year period. An innovation to Chinese exports to Japan uniformly leads to persistently lower levels of bilateral trade as well as relations, though the long-run effect on Chinese exports to Japan is only slightly lower than is the initial level.

The China-Korea case stands out uniquely from all others in the sense that a positive shock to Chinese exports to Korea produces much stronger relations while having a modest effect on trade in both directions (after some initial fluctuations).

The innovation to Chinese exports to Russia declines immediately if gradually after it occurs and reaches a new, persistent level within 10 months. Russian exports to China decline slightly before growing gradually. Relations improve then decline slightly.

The innovation to Chinese exports to India declines quickly but to a persistently higher level than initially. Indian exports to Chinese decrease sharply after 1 month but grow and surpass their initial level. Interestingly, relations between the two economies decline gradually and remain below initial levels.

Chinese-British relations improve modestly if persistently after a positive shock to Chinese exports to the UK while bilateral trade declines slightly, more so for British exports than for Chinese exports. The China-Australia relation demonstrates similar behavior though relations also remain lower than previously.

Chinese exports to France remain persistently higher after an innovation while French exports to China remain mostly lower than before. Relations exhibit few changes. This pattern also describes the China-Germany case though Chinese exports to Germany fall by a larger magnitude than do Chinese exports to France.

A positive shock on Chinese exports to Pakistan produces almost no effect on any other variables except Chinese exports to Pakistan. This effect disappears quickly and even leads to a persistent decline within 4 months. The China-Indonesia case is similar.

### Granger causality

Given that all country pairs that we examine contain at least 1 cointegrating relationship, we perform Granger Causality tests following a VAR on the levels of the variables. See Tables 1, 2 and 3 for a summary of the results. A “Y” indicates that we can reject the null hypothesis of no causality between row-variable (or variables for “All”) and the variable indicated in the table title.

**Table 1:** Granger Causality: Relations with China

	Australia	France	Germany	India	Indonesia	Japan	Korea	Pakistan	Russia	UK	US	Vietnam
Partner exports to China			Y									
Chinese exports to Partner					Y		Y			Y	Y	
Partner GDP			Y				Y				Y	
Chinese GDP		Y					Y					Y
All	Y				Y		Y		Y	Y	Y

**Table 2:** Granger Causality: Exports to China

	Australia	France	Germany	India	Indonesia	Japan	Korea	Pakistan	Russia	UK	US	Vietnam
Relations	Y	Y			Y		Y	Y				
Chinese exports to Partner	Y		Y	Y		Y	Y		Y		Y	
Partner GDP	Y	Y		Y	Y	Y	Y	Y	Y		Y	
Chinese GDP	Y	Y	Y		Y	Y	Y		Y	Y	Y	Y
All	Y	Y	Y	Y	Y	Y	Y	Y	Y	Y	Y	Y

**Table 3:** Granger Causality: China’s exports to Partner

	Australia	France	Germany	India	Indonesia	Japan	Korea	Pakistan	Russia	UK	US	Vietnam
Relations	Y	Y		Y	Y				Y	Y		
Partner exports to China	Y		Y		Y	Y	Y		Y	Y	Y	
Partner GDP	Y	Y		Y	Y		Y	Y	Y		Y	Y
Chinese GDP	Y	Y	Y	Y	Y	Y	Y	Y	Y	Y	Y	Y
All	Y	Y	Y	Y	Y	Y	Y	Y	Y	Y	Y	Y

Overall, political relations appear to Granger cause trade in either direction. Five countries demonstrate causality from their relations with China to their exports to China. Six countries demonstrate causality from relations to China’s exports to the countries. However, trade seems not to Granger cause changes in political relations. Even the results for the null hypothesis that all other variables in the VAR fail to Granger cause political relations are rejected for, at most, 6 of the 12 pairs. Chinese-Korean relations appear to be the relations most easily-explained by our model’s variables. In contrast, bilateral trade is easily explained by relations and previous bilateral trade.

### Multilateral VAR

As we stated in “Model specification” section, our previous estimating framework could not address the possibility for trade or political relations between China and country *j* to affect trade or relations between China and country *k* for *j*≠*k* (as would have been possible in a gravity model). This section analyzes results for a VAR where we include 11 of the 12 country pairs that we study to account for the possibility that relations between China and country *j* might affect trade or relations between China and country *k* for *j*≠*k*. We exclude India as data for India are not available for enough months contemporaneously with all other countries. Our main finding is no cointegrating vector for the 11 country pairs. Consequently, we estimate a VAR model in differences with 1 lag (as indicated by results from the SBIC).

Tables 4, 5 and 6 summarize the fully multilateral VAR results (See the full results in appendix 2). The results show some instances where 3^*rd*^ country effects can influence China’s trade and political relations with a particular country. Generally speaking, China’s foreign trade (especially exports from China) with country *j* have at least one statistically significant relationship with the lag of China’s trade with country *k*≠*j*. Trade between China and country *j* rarely depends on relations between China and country *k*≠*j*. In contrast, there are fewer instances where political relations between China and some country *j* have a statistically significant association with some country *k*≠*j*.

**Table 4:** Fully multilateral VAR results: Exports to China

	Australia	France	Germany	Indonesia	Japan	Korea	Pakistan	Russia	US	UK	Vietnam
Australia		From China (-)						From China (+)			
France								Relations (+)	From China (-)	Relations (+)	
Germany	To China (+)				From China (+)	From China (-)					
Indonesia											
Japan	To China (+)	Relations (+)	To China (+)					To China (-)	From China (-)	To China (+)	From China (-)
											Relations (-)
Korea	To China (+)		To China (+)		From China (+)				From China (-)		To China (+)
											Relations (-)
Pakistan								To China (+)			
Russia	To China (+)		To China (+)		From China (+)				From China (-)		
US	To China (+)	From China (-)		From China (+)	Relations (-)					Relations (-)	
UK	To China (+)						From China (+)	Relations (-)			
Vietnam	To China (+)		To China (+)			To China (-)		To China (-)		Relations (-)	
	From China (-)										
	Relations (-)										

Each row in this table corresponds to a single equation in the VAR system for a relation between China and the country identified in the lefthand side column. The nature of the relation (trade or political) is stated in the title of the table. A non-empty cell in the table denotes a statistically-significant (*p*-value ≤0.05) finding between a variable involving China and a country other than the country indicated by the entry in the lefthand side column of the row in question. The nature of the variable is indicated by the contents of the cell (“to China” means exports to China, “from China” means imports from China, “relations” means the political relations). The country with which China has this particular relation is stated at the top of the column of the cell in question

**Table 5:** Fully multilateral VAR results: Exports from China

	Australia	France	Germany	Indonesia	Japan	Korea	Pakistan	Russia	US	UK	Vietnam
Australia		From China (-)			To China (+)						To China (+)
France	To China (+)				To China (+)	To China (-)			From China (-)	To China (+)	
Germany	To China (+)				To China (+)	From China (-)	To China (+)	From China (+)			
Indonesia						To China (-)					
Japan	To China (+)	From China (-)	From China (+)						To China (+)		
									From China (-)		
Korea	To China (+)				From China (+)				To China (+)	To China (+)	To China (+)
									From China (-)		
Pakistan				To China (+)	To China (+)	To China (-)					
Russia		From China (-)			To China (+)				From China (-)		
US	To China (+)				To China (+)	To China (-)					
UK	To China (+)	From China (-)			To China (+)	To China (-)					
Vietnam	To China (+)	Relations (+)	Relations (-)	To China (+)	To China (+)	To China (-)			From China (-)	To China (+)	
	Relations (-)				From China (+)						

Each row in this table corresponds to a single equation in the VAR system for a relation between China and the country identified in the lefthand side column. The nature of the relation (trade or political) is stated in the title of the table. A non-empty cell in the table denotes a statistically-significant (*p*-value ≤0.05) finding between a variable involving China and a country other than the country indicated by the entry in the lefthand side column of the row in question. The nature of the variable is indicated by the contents of the cell (“to China” means exports to China, “from China” means imports from China, “relations” means the political relations). The country with which China has this particular relation is stated at the top of the column of the cell in question

**Table 6:** Fully multilateral VAR results: Relations with China

	Australia	France	Germany	Indonesia	Japan	Korea	Pakistan	Russia	US	UK	Vietnam
Australia									To China (+)		From China (+)
France	Relations (+)										
Germany	To China (+)				Relations (-)		To China (+)				To China (-)
Indonesia						From China (+)		From China (+)			
Japan										To China (+)	
Korea				To China (-)			To China (+)	From China (+)		Relations (+)	
Pakistan			From China (-)	From China (-)		From China (+)					
Russia			To China (-)				From China (+)		Relations (-)		To China (+)
US											
UK									From China (+)		
Vietnam	From China (+)	From China (+)	From China (-)					From China (+)	From China (-)		

Each row in this table corresponds to a single equation in the VAR system for a relation between China and the country identified in the lefthand side column. The nature of the relation (trade or political) is stated in the title of the table. A non-empty cell in the table denotes a statistically-significant (*p*-value ≤0.05) finding between a variable involving China and a country other than the country indicated by the entry in the lefthand side column of the row in question. The nature of the variable is indicated by the contents of the cell (“to China” means exports to China, “from China” means imports from China, “relations” means the political relations). The country with which China has this particular relation is stated at the top of the column of the cell in question

A few patterns emerge from the result. Chinese-Indonesian political relations and trade flows are the least influenced by 3^*rd*^ parties while trade between China and each of Vietnam, Japan, and Korea are seemingly the most responsive to China’s links with other countries. The importance of these three countries may be a consequence of long-standing political, economic, and cultural links.

Interestingly, a few patterns emerge when examining regressors across equations rather than individual equations. Australian-Chinese trade frequently appears as a positive determinant for Chinese trade in both directions with other economies (7 equations each for exports to and from China). Japanese exports to China are a positive determinant for exports from China to 8 different economies while Korean exports to China are a positive determinant for exports from China to 6 countries. These patterns may emerge from production chains where raw materials (harvested in Australia) and/or intermediate goods (produced in Japan and Korea) are key inputs in China’s exports to the rest of the world.

## Conclusion and further discussion

Conventional wisdom dictates that with a greater volume of international trade and a greater degree of international economic dependence, countries tend to be more restrained in international conflicts. In turn, under a more peaceful and harmonious environment, consumers and businesses tend to have less “home bias” in their purchases of goods and services. While proponents of greater international economic integration have cited such conventional wisdom to ensure global stability, recent events (e.g., Brexit, COVID-19 pandemic) have caused people to think otherwise. Our paper provides an empirical test of the conjecture that the warming and cooling of political ties between two countries is accompanied by expansion and contraction of their trade activities with each other.

To perform the empirical investigation of the interdependence between economic and political spheres, we employ data of political relations and trade flows between China and her twelve large trading partners during the period of 1981–2019. We find that political, relations, and GDP exist in a steady-long run relationship as evidenced by the existence of cointegrating vectors among the variables. We find that shocks to political relations variables are persistent while shocks to export variables are less persistent. Granger causality tests indicate that relations Granger cause trade more often than do trade flows Granger cause relations. While trade and political relationships usually demonstrate a positive relationship (as indicated by the IRFs), several cases of a negative relationship between the variables do exist.

Owing to recent political tension at the time of this paper’s writing, a closer examination into the US-Chinese case is instructive. The IRF for US-Chinese political relations shows a high level of persistence: when relations improve, they stay improved for a long time. The counterpart, of course, is that if relations worsen, then they stay worsened for a long time. Trade, however, demonstrates little reaction in response to a change in relations. A positive shock to trade, however, yields different results depending on the type of trade flow. A positive shock to China’s exports to the US leads to persistently higher levels of exports to the US, US exports to China, and relations (measured from China’s perspective). Yet, a positive shock to America’s exports to China leads to persistently *worse* relations with China while trade in both directions increases slightly. If these responses implied by the IRFs indicate common patterns based on years of data, then it stands to reason that Chinese officials genuinely do feel that China’s relations with the US worsen when the US increases its exports to China. Only one other country, South Korea, exhibits a worsening in relations with China, similar in magnitude to that of relations with the US, following growth in exports to China. A logical consequence of worsening relations would be that Chinese officials would undertake measures to reduce US exports to China. Consequently, claims made by US officials that the Chinese government seeks to limit non-Chinese (or at least American) exports into China may have some merit^11^.

The managerial implication of our study is that although intergovernmental or interstate amity and enmity are usually out of control of businesses, companies still need to prepare for instability caused by changing political and diplomatic climate. Given that political relationship can lead to intended and unintended consequences for manufacturers and trading companies, operations leaders have a responsibility to put their companies in a position to capitalize on new opportunities for business creation and growth while avert the damages caused by potential political risks. Importers should enhance their adaptive capabilities into their supply chain strategies and shipping management, and exporters should better align demand and supply and develop viable response plans when adjusting their marketing strategies. Since the induced changes in international trade may further cause a shift in global supply chain, logistics and shipping companies, which usually tend to pay much more attention on cost, time and reliability of delivery than on political factors that shape the external business environment, should be aware of international political relationship that may lead to the evolution of shipping networks.

While our paper tests the effect of China’s foreign relations on its trade activities, the conjecture can be applied to other contexts. In 2012, Nobel Peace Prize was awarded to the European Union (EU) “*for over six decades contributed to the advancement of peace and reconciliation, democracy and human rights in Europe.*”^12^ The EU’s main practical function has been promoting international trade among its member countries. The EU’s achievements belong to a larger set of greater international, political and economic integration witnessed in the post-World War II era, which provides evidence that better political relations and a greater volume of international trade go hand-in-hand. In contrast, there are numerous events that adversely affect interstate political relations in the EU. Recent examples include political disagreement between the United Kingdom and other member states of the European Union over immigration and refugee policy. Despite the deep economic links created by trade between the United Kingdom and other EU members, this disagreement led to the Brexit in January 2020 and an anticipated decline in UK-European trade.

## Data Availability

The datasets used and/or analysed during the current study and the two appendices are available upon request.

## Footnotes

1 See “National Security Strategy of the United States of America”, The White House, Washington DC, US, December 2017.

2 See “Coronavirus solidifies US-China decoupling” by Christina Lin, *Asian Times*, February 25, 2020.

3 See “Boycott hits Toyota” by Emily, F. *Times* (The United Kingdom), 2012 p. 56. “Sleepy islands and a smoldering dispute” by Fackler, M. and Johnson, I. *New York Times*, September 21, 2012. “Japan exports plummet as Chinese orders dry up.” by Lynch, R. *Evening Standard* (The United Kingdom), 2012.

4 See http://www.imir.tsinghua.edu.cn/publish/iis/7522/index.

5 See http://www.imir.tsinghua.edu.cn/publish/iis/7522/20120522140122561915769/1.pdf(in Chinese only). Translation by authors.

6 Translation by authors.

7 See “For Taiwan, new access to Western arms” by S. Wu Dunn, *New York Times*, September 24, 1991. “China warns Sarkozy not to see Dalai Lama.” by S. Erlanger, *New York Times*, July 9, 2008.

8 See “Dustbin lids used as shields” by P. Hopkirk, *The Times*, August 30, 1967; “Peking demonstrators set fire to British embassy buildings.” by J. Vincent. *The Times*, August 23, 1967; “Peking takes credit for deal” by D. Bonavia. 1984. *The Times*, 28 August 28, 1984.

9 See “Japan-China island tensions rise.” by M. Ito, *Japan Times*, 2010(a). “Trawler’s collisions, jcg arrest of skipper near senkakus protested.” by M. Ito, *Japan Times*, 2010(b). “Governor seen as goading administration into action.” by M. Ito, and J. Hongo, *Japan Times*, 2012. “Panetta tells Japan, China to resolve Senkaku row peacefully.” by J. Hongo, *Japan Times*, 2012.

10 See “Indian minister mollifies China: London edition.” by M. Nicholson, *Financial Times*, May 7, 1998.

11 See “Barrier to entry - China’s restrictions on U.S. imports,” *Reuters*, March 14, 2018 and *How China’s Economic Aggression Threatens the Technololgies and Intellectual Property of the United States and the World*, White House Office of Trade and Manufacturing Policy, June 2018, https://www.whitehouse.gov/wp-content/uploads/2018/06/FINAL-ChinaTechnology-Report-6.18. 18-PDF.pdf

12 See “The Nobel Peace Prize 2012 to the European Union (EU) – Press Release.” Nobel Media AB 2014. Web. 24 Oct 2016.
